# Glutathione Synthesis Regulated by CtrA Protects *Ehrlichia chaffeensis* From Host Cell Oxidative Stress

**DOI:** 10.3389/fmicb.2022.846488

**Published:** 2022-03-30

**Authors:** Jiaqi Yan, Qi’an Liang, Zhouyi Chai, Nan Duan, Xiaoxiao Li, Yajing Liu, Nan Yang, Meifang Li, Yongxin Jin, Fang Bai, Weihui Wu, Zhihui Cheng

**Affiliations:** Key Laboratory of Molecular Microbiology and Technology of the Ministry of Education, Department of Microbiology, College of Life Sciences, Nankai University, Tianjin, China

**Keywords:** *Ehrlichia chaffeensis*, human monocytic ehrlichiosis, GshA, GshB, CtrA

## Abstract

*Ehrlichia chaffeensis*, a small Gram-negative obligatory intracellular bacterium, infects human monocytes or macrophages, and causes human monocytic ehrlichiosis, one of the most prevalent, life-threatening emerging zoonoses. Reactive oxygen species are produced by the host immune cells in response to bacterial infections. The mechanisms exploited by *E. chaffeensis* to resist oxidative stress have not been comprehensively demonstrated. Here, we found that *E. chaffeensis* encodes two functional enzymes, GshA and GshB, to synthesize glutathione that confers *E. chaffeensis* the oxidative stress resistance, and that the expression of *gshA* and *gshB* is upregulated by CtrA, a global transcriptional regulator, upon oxidative stress. We found that in *E. chaffeensis*, the expression of *gshA* and *gshB* was upregulated upon oxidative stress using quantitative RT-PCR. *Ehrlichia chaffeensis* GshA or GshB restored the ability of *Pseudomonas aeruginosa* GshA or GshB mutant to cope with oxidative stress, respectively. Recombinant *E. chaffeensis* CtrA directly bound to the promoters of *gshA* and *gshB*, determined with electrophoretic mobility shift assay, and activated the expression of *gshA* and *gshB* determined with reporter assay. Peptide nucleic acid transfection of *E. chaffeensis*, which reduced the CtrA protein level, inhibited the oxidative stress-induced upregulation of *gshA* and *gshB*. Our findings provide insights into the function and regulation of the two enzymes critical for *E. chaffeensis* resistance to oxidative stress and may deepen our understanding of *E. chaffeensis* pathogenesis and adaptation in hosts.

## Introduction

*Ehrlichia chaffeensis* is a small Gram-negative obligatory intracellular bacterium that preferentially infects human monocytes or macrophages and causes human monocytic ehrlichiosis (HME), one of the most prevalent, life-threatening emerging zoonoses (Rikihisa, 2010). Patients with HME exhibit symptoms including headache, fever, myalgia, and malaise, and hematological abnormalities including anemia, leucopenia, thrombocytopenia, and elevated hepatic aminotransferases (Ismail et al., 2010). The number of HME cases reported in the United States was 2,093 in 2019, showing a more than 10-fold increase over a 10-year period (CDC, 2021). Approximately 40–60% of patients require hospitalization, and the estimated case fatality rate for HME is approximately 3% (Paddock and Childs, 2003). Studies on the mechanisms exploited by *E. chaffeensis* to cope with host immune responses may assist in understanding the pathogenesis of HME and discovering the next-generation HME treatments.

When hosts encounter pathogenic bacteria, the NADPH oxidase complex of host phagocytes assembles and generates reactive oxygen species (ROS; Teymournejad et al., 2017; Sies and Jones, 2020), which destroy the integrity of cell membranes and oxidize DNA and proteins (Puppo et al., 2005). *Ehrlichia chaffeensis* induces the degradation of p22^phox^, a component of the NADPH oxidase complex in infected macrophages and inhibits the recruitment of NADPH oxidase components to bacterial replicative inclusions (Lin and Rikihisa, 2007). *Ehrlichia chaffeensis* EtpE blocks the generation of ROS in a CD147-dependent way, and Etf-1 upregulates host MnSOD to reduce ROS levels in macrophages (Liu et al., 2012; Teymournejad and Rikihisa, 2020). However, *E. chaffeensis* cannot inhibit ROS production in human neutrophils (Lin and Rikihisa, 2007). *Ehrlichia chaffeensis* encodes FeSOD and AhpC, which have been suggested to reduce ROS levels in macrophages (Ohashi et al., 2002; Dunning Hotopp et al., 2006); however, their functions have not been demonstrated. Thus, studies on the mechanisms exploited by *E. chaffeensis* to respond to the oxidative stress will provide new information in understanding the adaptation of *E. chaffeensis* in hosts.

Glutathione (GSH), a tripeptide (γ-glutamylcysteinylglycine), is the most potent natural antioxidant that directly or indirectly eliminates ROS (Smirnova and Oktyabrsky, 2005; Morris et al., 2013). Glutamate-cysteine ligase (GshA) catalyzes glutamate and cysteine to generate γ-glutamylcysteine, then glutathione synthetase (GshB) adds glycine to generate GSH (Smirnova and Oktyabrsky, 2005). The role of GSH in pathogenesis has been demonstrated by the results that *Salmonella enterica* serovar Typhimurium lacking *gshA* is attenuated in the acute model of *Salmonella* infection (Song et al., 2013). *Pseudomonas aeruginosa* lacking GSH production shows increased oxidative sensitivity, and reduced swimming and swarming motilities (Van Laar et al., 2018; Zhang et al., 2019). In *E. chaffeensis*, ECH_0125 (GenBank ID: ABD45326) and ECH_0336 (GenBank ID: ABD44941) encode GshA and GshB, respectively. However, whether these two enzymes are functional in *E. chaffeensis* and confer bacteria the ability to resist oxidative stress are still unknown.

In *Sinorhizobium meliloti*, glutathione synthesis is regulated by LsrB and OxyR (Lu et al., 2013), as well as by the two-component regulatory system (TCS) ActS/ActR to adapt to oxidative stress (Tang et al., 2017). However, *E. chaffeensis* lacks homologs of these genes (Dunning Hotopp et al., 2006). CtrA is a global transcriptional regulator that recognizes the consensus 8-mer binding motif (TTAACCAT) and the 9-mer binding motif (TTAAN_7_TTAAC; Quon et al., 1996; Laub et al., 2002). In *E. chaffeensis*, CtrA upregulates the expression of genes that confer the bacterium resistance to physicochemical stresses, including oxidative stress (Cheng et al., 2011). In this study, to gain insights into the roles of GshA and GshB in *E. chaffeensis* intracellular infection, we determined their function using a *P. aeruginosa* surrogate system and illustrated the mechanism regulating their expression by CtrA using peptide nucleic acid (PNA) transfection, electrophoretic mobility shift assays (EMSAs), and enhanced green fluorescent protein (EGFP) reporter assays in *Escherichia coli.*

## Materials and Methods

### Bacteria Strains and Culture, Plasmids, and Primers

*Ehrlichia chaffeensis* Arkansas strain was cultured in human acute leukemia THP-1 cell line in RPMI 1640 medium supplemented with 2 mM L-glutamine and 10% fetal bovine serum (FBS; Tianhang, Zhejiang, China) at 37°C in 5% CO_2_ and 95% air, as described previously (Cheng et al., 2008). *Escherichia coli* strains DH5α and BL21 (DE3) for DNA cloning and protein expression were cultured at 37°C in Luria–Bertani (LB) broth supplemented with appropriate antibiotics (50 μg/ml of kanamycin, 100 μg/ml of ampicillin, or 34 μg/ml of chloramphenicol) when necessary (Cheng et al., 2011). *Pseudomonas aeruginosa* strains were cultured at 37°C in LB broth supplemented with 150 μg/ml of carbenicillin or 50 μg/ml of gentamicin when necessary (Weng et al., 2016).

The plasmids used in this study are listed in Supplementary Table S1. For DNA manipulation, standard protocols or manufacturer instructions of commercial products were followed. The primers used for gene cloning, EMSA, and qRT-PCR are listed in Supplementary Table S1.

### Isolation of Host Cell-Free *Ehrlichia chaffeensis*

*Ehrlichia chaffeensis* was isolated from infected THP-1 cells as described previously (Teymournejad and Rikihisa, 2020). Briefly, *E. chaffeensis*-infected THP-1 cells (~2 × 10^7^ cells, >95% infected) were harvested at 600 × *g* at room temperature for 5 min. The pellet was suspended in fresh culture medium and passed through a 23-gauge needle with a syringe on ice for 20 times to crush the host cell membrane. To remove unbroken cells and cell debris, the mixture was centrifuged at 1,000 × *g* at 4°C for 5 min. The supernatant was collected by additional centrifugation at 10,000 × *g* at 4°C for 10 min. The bacterial pellet was suspended in fresh culture medium for synchronous culture or in 0.3 M sucrose for PNA transfection.

### Expression and Purification of Recombinant Proteins

The DNA fragments encoding full-length CtrA and Tr1 were cloned into pET-33b(+) for recombinant CtrA (rCtrA) and recombinant Tr1 (rTr1), pET-41a(+) for GST-rCtrA as described previously (Cheng et al., 2006). *Escherichia coli* BL21 (DE3) cells were transformed with plasmids to express the recombinant proteins. The strains were induced to express rCtrA with 1 mM isopropyl-thio-β-D-galactoside (IPTG; Solarbio, Beijing, China) at 37°C for 4 h, or to express rTr1 with 1 mM IPTG at 20°C for 5 h, or to express GST and GST-rCtrA with 0.1 mM IPTG at 37°C for 4 h. rCtrA and rTr1 were purified from *E. coli* inclusion bodies using 6 M urea, and GST and GST-rCtrA were purified from *E. coli* soluble fraction (Cheng et al., 2006). The purified proteins were dialyzed against stocking buffer [10 mM Tris-HCl (Genview, Beijing, China), pH 7.5, 1 mM dithiothreitol (DTT; Solarbio)] for further experiments.

### Antibody Preparation

Antibodies against *E. chaffeensis* CtrA and Tr1 were prepared as described previously (Cheng et al., 2006). Purified rCtrA and rTr1 were cut from the SDS-PAGE gel, then sent to ABclonal Biotechnology Co., Ltd. (Wuhan, China) for rabbit polyclonal antibody preparation. Purified rCtrA or rTr1 was injected into Japanese white rabbits four times every 2 weeks. Twelve days after the fourth injection, 20 ml of total blood was collected from the rabbits to prepare the antiserum.

### Peptide Nucleic Acid Transfection

Peptide nucleic acid transfection to knockdown CtrA in *E. chaffeensis* was performed as described previously (Yan et al., 2021). An antisense PNA oligomer targeting 31–46 bp following the start codon of *ctrA* (CtrA PNA) 3′-CGTACACGTTTCCGTC-5′ and a control PNA (CTL PNA) 3′-CACATATCTCGG-5′ were synthesized by KareBay™ Biochem, Inc. (Ningbo, China). Three micrograms of CtrA PNA or CTL PNA dissolved in nuclease-free water was mixed with 100 μl of host cell-free *E. chaffeensis* in 0.3 M sucrose, then incubated on ice for 15 min. Electroporation was conducted at 2,000 V, 25 μF, and 400 Ω with a 10-ms pulse using a Gene Pulser Xcell™ electroporation system (Bio-Rad, Hercules, CA, United States) in a 2-mm electroporation cuvette (Bio-Rad). Then the PNA-transfected *E. chaffeensis* was transferred to a T25 flask to infect 5 × 10^5^ THP-1 cells and incubated at 37°C for 2 h with gentle shaking every 15 min to facilitate bacterial internalization. To detect the effect of CtrA PNA, infected cells were harvested at 36 h p.i. The expression level of *ctrA* and the protein level of CtrA were examined by qRT-PCR and Western blotting, respectively.

### H_2_O_2_ Assay

The susceptibility of *P. aeruginosa* to H_2_O_2_ was determined as described previously (Weng et al., 2016). Overnight-cultured *P. aeruginosa* strains were diluted in LB broth to an OD_600_ of 0.03 and cultured at 37°C. At an OD_600_ of 1.0, bacteria from 1 ml of culture were collected and washed three times with 1× PBS. The pellet was resuspended in 1× PBS and treated with H_2_O_2_ at a final concentration of 50 mM at 37°C for 15 min. The number of live bacteria was determined by gradient dilution and plate counting.

*Ehrlichia chaffeensis* was treated with H_2_O_2_ as described previously (Yen et al., 2020). THP-1 cells were synchronously infected with host cell-free *E. chaffeensis*, CTL PNA-, or CtrA PNA-transfected *E. chaffeensis*. At 36 h p.i., 20 μl of 10 mM H_2_O_2_ diluted in culture medium was added into 2 ml of the synchronous culture, reaching a final concentration of 100 μM. After 2 h of treatment at 37°C, the expression levels of *gshA* and *gshB* were determined with qRT-PCR.

### Quantitative RT-PCR

Total RNA was extracted from each sample and reverse transcribed to cDNA as described previously (Duan et al., 2021). The amounts of *E. chaffeensis* 16S rRNA, *ctrA*, *gshA*, *gshB*, *p28*, and human *GAPDH* were determined with qRT-PCR using specific primers (Supplementary Table S1) and the ChamQ Universal SYBR qPCR Master Mix (Vazyme, Nanjing, China) on a StepOnePlus™ Real-Time PCR System (Applied Biosystems, MA, United States). The expression levels of the relative genes were normalized against that of *E. chaffeensis* 16S rRNA. Bacterial growth was determined as the amount of bacterial 16S rRNA normalized against that of human *GAPDH* mRNA.

### Swimming and Swarming Motility Assays

Swimming and swarming motilities were performed as described previously (Zhang et al., 2019). Swimming media consisting of 0.3% agar was supplemented with 10 g/L of tryptone and 5 g/L of NaCl (pH 7.2). Swarming media consisting of 0.35% agar was supplemented with 62 nM K_3_PO_4_, 2 nM MgSO_4_, 10 μM FeSO_4_, 0.4% glucose, and 0.1% casein hydrolysate. *Pseudomonas aeruginosa* strains were cultured overnight at 37°C and then diluted to an OD_600_ of 0.1. Two microliters of the diluted bacterial solution was center spotted onto the surface of the corresponding agar plates. The swimming plates were incubated at 30°C for 16 h, while the swarming plates were incubated at 37°C for 14 h. Bacterial motilities were imaged using a ChemiDoc™ XRS+ camera (Bio-Rad) and assessed by measuring the diameter of the widest point of spread on each plate. All experiments were repeated five times for data analysis.

### Electrophoretic Mobility Shift Assay

Electrophoretic mobility shift assay was performed as described previously (Cheng et al., 2011). Briefly, the promoter regions of *gshA* (391 bp), *gshB* (460 bp), and *p28* (260 bp) were amplified with PCR using the specific primers (Supplementary Table S1). The purified GST-rCtrA (2.5 μM) was incubated with 50 ng of DNA probes in a 20-μl reaction mixture containing 10 mM Tris-HCl (pH 7.5), 1 mM DTT, and 1% glycine (Solarbio) on ice for 30 min. Samples were loaded onto 8% native polyacrylamide gel in 1× TBE buffer, which had been prerun for 1 h at 100 V, and electrophoresed at 10 mA on ice for 1.5 h. The gel was stained in 1× TBE containing 0.5 μg/ml ethidium bromide at room temperature for 10 min. Bands were visualized using a molecular imager ChemiDoc™ XRS+ (Bio-Rad).

### Construction of Enhanced Green Fluorescent Protein Fusions and Reporter Assay

Enhanced green fluorescent protein fusions were constructed as described previously (Duan et al., 2021). Briefly, the promoter region of *gshA*, *gshB*, or *p28* was amplified and inserted upstream of the promoter-less *egfp* gene in the pQE60 vector. *Escherichia coli* BL21 (DE3) strain containing pACYCDuet-1 harboring *ctrA* (pACYCDuet-1-rCtrA) or the empty pACYCDuet-1 vector was transformed with the pQE60–promoter–EGFP fusion constructs. After induction of rCtrA expression with 0.05 mM IPTG at 37°C for 3 h, bacterial samples were collected and subjected to Western blotting to measure the amounts of EGFP, RpoA, and rCtrA in each sample.

### Western Blotting

To detect the CtrA protein levels after PNA transfection, CTL PNA- or CtrA PNA-transfected *E. chaffeensis*-infected THP-1 cells were harvested by centrifugation at 500 × *g* for 5 min at 36 h p.i. The pellet was suspended in 1× PBS and immediately sonicated. The samples were then subjected to 12% SDS-PAGE, transferred to a PVDF membrane, and incubated with anti-CtrA or anti-Tr1 rabbit antiserum, respectively. After being washed, the membranes were incubated with secondary HRP-conjugated goat anti-rabbit IgG (Promega, WI, United States, W401B). The specific bands were detected with an Immobilon Western kit (Millipore, MA, United States) and a molecular imager ChemiDoc™ XRS+. The relative amount of CtrA or Tr1 in CtrA PNA-transfected *E. chaffeensis* was normalized against that in CTL PNA-transfected *E. chaffeensis*, respectively.

To detect the amount of EGFP in the reporter assay, an equivalent number of *E. coli* cells was collected. The protein levels of EGFP, RpoA, and rCtrA were determined using a mouse monoclonal anti-GFP antibody (GeneTex Inc., North America, GTX628528), mouse monoclonal anti-*E. coli* RNA polymerase α antibody (BioLegend, San Diego, CA, United States, 663104), or specific rabbit polyclonal anti-CtrA antiserum, respectively. The relative amounts of EGFP or RpoA in strains expressing rCtrA were normalized against those in strains containing pACYCDuet-1 vector.

### Statistical Analysis

All experiments were repeated at least three times. Statistical analyses were performed using GraphPad Prism 7.0. Statistical significance of a two-group comparison was assessed using Student’s *t*-test (two-tailed). A value of *p* < 0.05 was considered significant.

## Results

### The Expression of *gshA* and *gshB* in *Ehrlichia chaffeensis* Is Upregulated Upon Oxidative Stress

**Figure 1:**
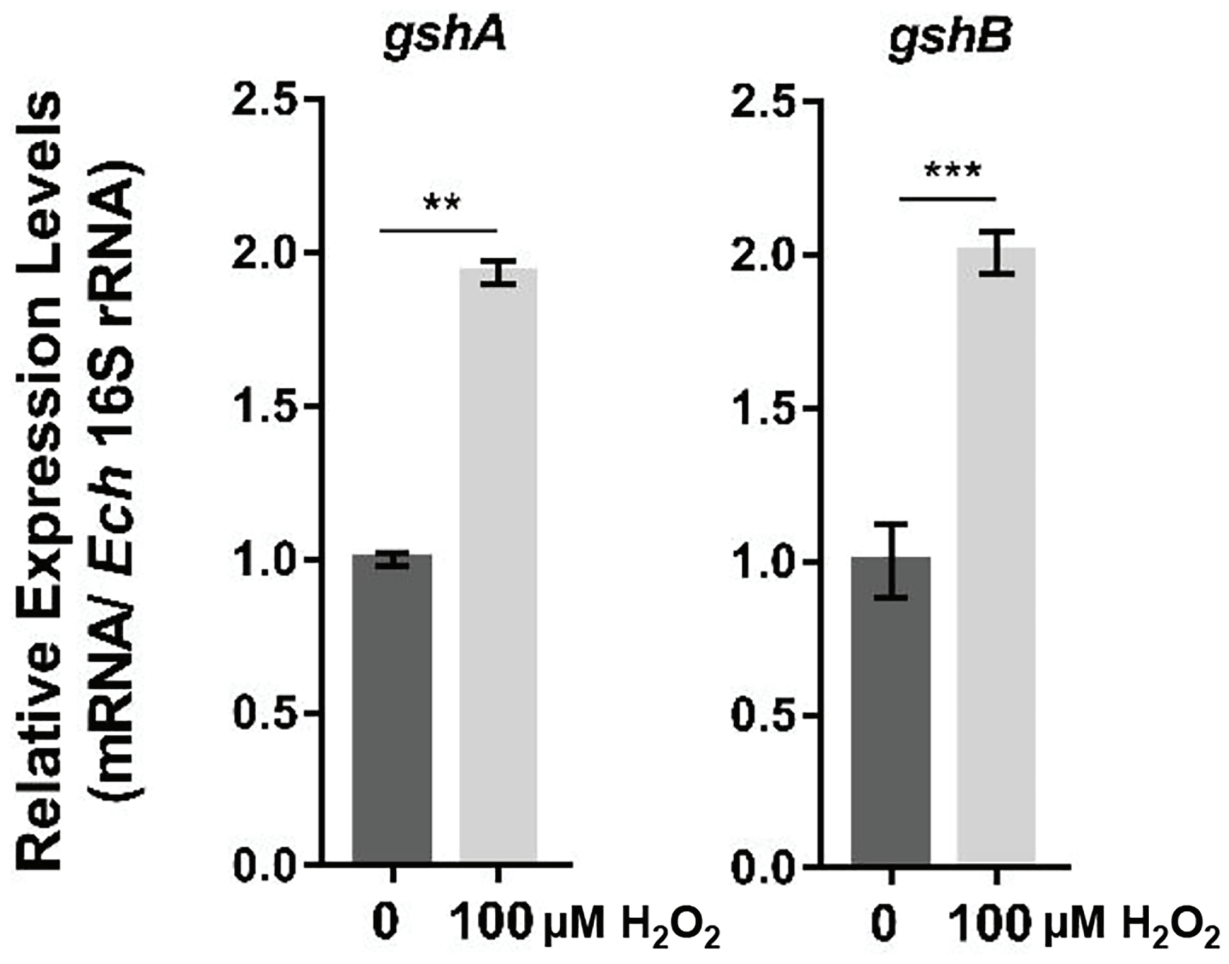
The expression of *gshA* and *gshB* is upregulated upon oxidative stress. At 36 h p.i. synchronously *E. chaffeensis*-infected THP-1 cells were treated with H_2_O_2_ at a final concentration of 100 μM or culture medium (CTL) at 37°C for 2 h. The expression levels of *gshA* and *gshB* were determined with qRT-PCR and normalized against those of *E. chaffeensis* 16S rRNA. The value of *gshA* or *gshB* in CTL sample is as 1, respectively. Relative values to the amount in CTL sample are shown. Data indicate means ± standard deviations (*n* = 3). The significant differences are represented by *p*-values determined with Student’s *t*-test (^**^p < 0.01, ^***^p < 0.001).

The production of ROS by host cells is one of the major mechanisms of host defense against bacterial infections (Ziltener et al., 2016). We first investigated whether GshA and GshB in *E. chaffeensis* are required for bacteria to respond to oxidative stress. At 36 h p.i., *E. chaffeensis*-infected THP-1 cells were treated with 100 μM H_2_O_2_ at 37°C for 2 h. We found that the expression of *gshA* and *gshB* was significantly upregulated after H_2_O_2_ treatment (Figure 1), suggesting that GshA and GshB might be involved in *E. chaffeensis* response to oxidative stress.

### *Ehrlichia chaffeensis* GshA or GshB Restores the Ability of *Pseudomonas aeruginosa* GshA or GshB Mutant to Cope With Oxidative Stress

**Figure 2:**
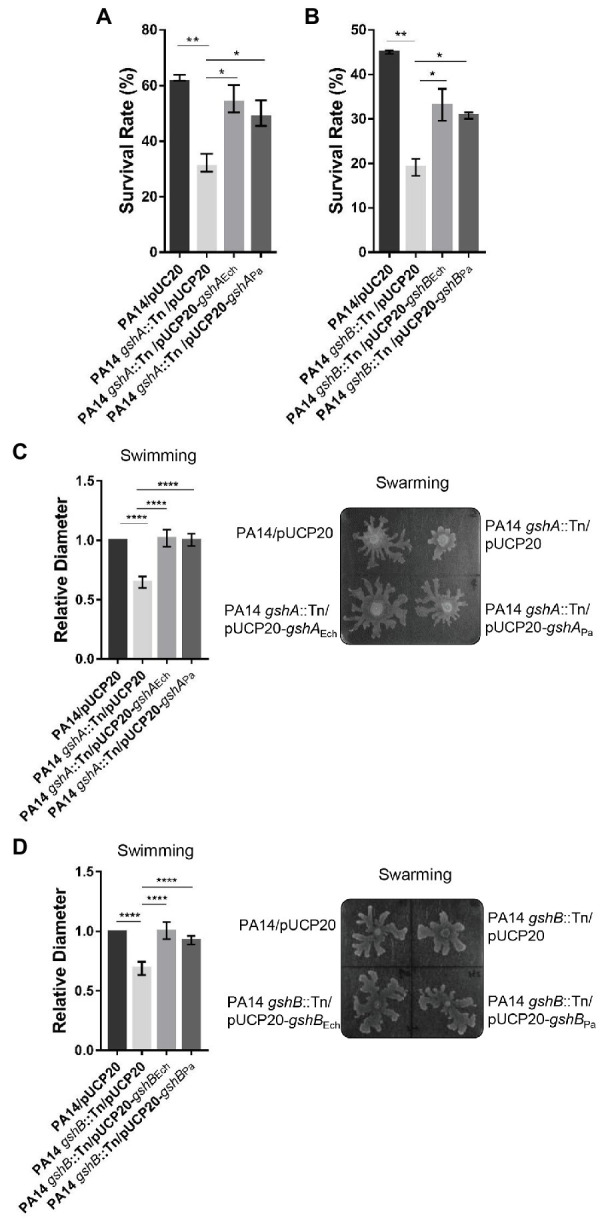
*Ehrlichia chaffeensis* GshA or GshB restores the survival ability under oxidative stress and the motility of corresponding *P. aeruginosa* mutant. **(A,B)** *Ehrlichia chaffeensis* GshA **(A)** or GshB **(B)** confers survival ability under oxidative stress to *P. aeruginosa* mutant. *Pseudomonas aeruginosa* strains grown to an OD_600_ of 1.0 were treated with H_2_O_2_ at a final concentration of 50 mM at 37°C for 15 min. The survival rate is shown as the ratio of the colony number of each strain to that of the control group. Data indicate means ± standard deviations (*n* = 3). The significant differences are represented by *p*-values determined with Student’s *t*-test (^*^p < 0.05, ^**^p < 0.01). **(C,D)** *Ehrlichia chaffeensis* GshA **(C)** or GshB **(D)** restores the swimming and swarming motilities of the corresponding *P. aeruginosa* mutant. Two microliters of diluted overnight culture (OD_600_ = 0.1) of each strain was center spotted onto respective culture plates and incubated at 30°C for 16 h or at 37°C for 14 h, respectively. Swimming motility was assessed by measuring the diameter of the widest point of spread on each plate. Relative values to the diameter of wild-type PA14 are shown. Data indicate means ± standard deviations (*n* = 5). The significant differences are represented by *p*-values determined with Student’s *t*-test (^****^p < 0.0001). Swarming motility was assessed five times with similar results. Images from one typical experiment are shown.

We then investigated the function of *E. chaffeensis* GshA and GshB. No *E. chaffeensis* deletion mutant of GshA or GshB is currently available, because classical bacteriology techniques, such as targeted mutagenesis, are not readily applicable for obligatory intracellular bacteria (McClure et al., 2017). To overcome this limitation, we used *P. aeruginosa* as a surrogate system to study the functions of *E. chaffeensis* GshA and GshB. In *P. aeruginosa*, GshA and GshB are critical for bacteria to resist oxidative stress (Van Laar et al., 2018; Zhang et al., 2019). The enzyme domain of *E. chaffeensis* GshA or GshB shows 55.0% or 64.7% identity to that of *P. aeruginosa* GshA or GshB, respectively (Supplementary Figure S1). We then expressed *gshA* or *gshB* from *E. chaffeensis* or *P. aeruginosa* in a *gshA*::Tn mutant or a *gshB*::Tn mutant of the *P. aeruginosa* reference strain PA14 (Liberati et al., 2006). The growth rates of the wild-type PA14, the GshA mutant, the GshB mutant, and the complemented mutants showed no difference (Supplementary Figures S2A,B). The bacteria were grown to the exponential phase and treated with 50 mM H_2_O_2_ at 37°C for 15 min. The survival rate of the *gshA*::Tn or the *gshB*::Tn mutant strain was significantly lower than that of the wild-type PA14 (Figures 2A,B), which was restored by the expression of *gshA* from *E. chaffeensis* or *P. aeruginosa* (Figure 2A), or by the expression of *gshB* from *E. chaffeensis* or *P. aeruginosa* (Figure 2B), respectively. These results indicate that *E. chaffeensis* GshA and GshB are functional and confer the ability of bacterial defense against oxidative stress, which aids *E. chaffeensis* in establishing infection in hosts.

Deletion of GshA or GshB affects *P. aeruginosa* swimming and swarming motilities due to the disruption in the redox status (Van Laar et al., 2018; Zhang et al., 2019). We then examined whether *E. chaffeensis* GshA or GshB can restore the swimming and swarming motilities of the *P. aeruginosa gshA*::Tn or *gshB*::Tn mutant, respectively. The swimming and swarming motilities of the *gshA*::Tn or the *gshB*::Tn mutant strain were significantly reduced compared with that of the wild-type PA14, which were restored by the expression of *gshA* from *E. chaffeensis* or *P. aeruginosa* (Figure 2C and Supplementary Figure S3A), or by the expression of *gshB* from *E. chaffeensis* or *P. aeruginosa* (Figure 2D and Supplementary Figure S3B), respectively. These results further confirm that *E. chaffeensis* GshA and GshB are functional.

### The Expression of *gshA* and *gshB* Is Regulated by CtrA in *Ehrlichia chaffeensis*

**Figure 3:**
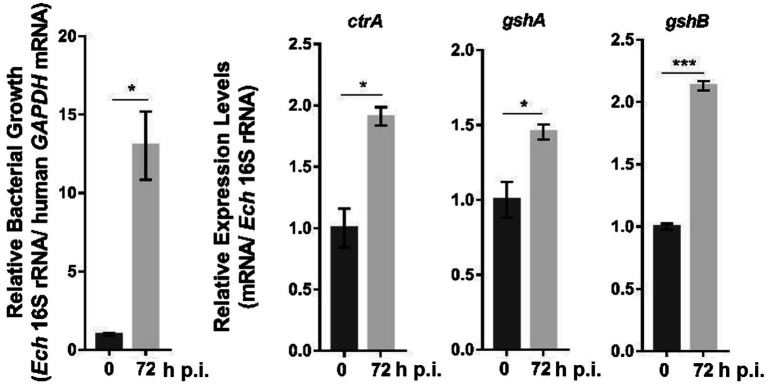
The expression of *ctrA*, *gshA*, and *gshB* at different stages of *E. chaffeensis* intracellular growth. RNA samples were prepared from synchronously cultured *E. chaffeensis* in THP-1 cells at 0 h and 72 h p.i. The expression levels of each gene were determined with qRT-PCR and normalized against those of *E. chaffeensis* 16S rRNA. Relative values to the amount at 0 h p.i. are shown. Data indicate means ± standard deviations (*n* = 3). The significant differences are represented by *p*-values determined with Student’s *t*-test (^*^p < 0.05, ^***^p < 0.001).

As GshA and GshB are functional in *E. chaffeensis*, we next investigated the mechanisms by which their expression is regulated. *Ehrlichia chaffeensis* CtrA upregulates the expression of genes that confer physicochemical stress resistance to bacteria (Cheng et al., 2011). We found one 8-mer binding motif in the *gshA* promoter region (−217 to −210 calculated from the translational start site) and one 9-mer binding motif containing a 1-bp mismatch in the *gshB* promoter region (−296 to −281 calculated from the translational start site). CtrA is expressed at the late stage of *E. chaffeensis* intracellular growth (Cheng et al., 2011). Using quantitative RT-PCR (qRT-PCR), we examined the expression patterns of *gshA* and *gshB* in synchronously cultured *E. chaffeensis* in THP-1 cells. After normalization against bacterial 16S rRNA, the expression of *gshA* and *gshB* was also upregulated at the late stage of *E. chaffeensis* intracellular growth (Figure 3), indicating that the expression of *gshA* and *gshB* might be regulated by CtrA.

**Figure 4:**
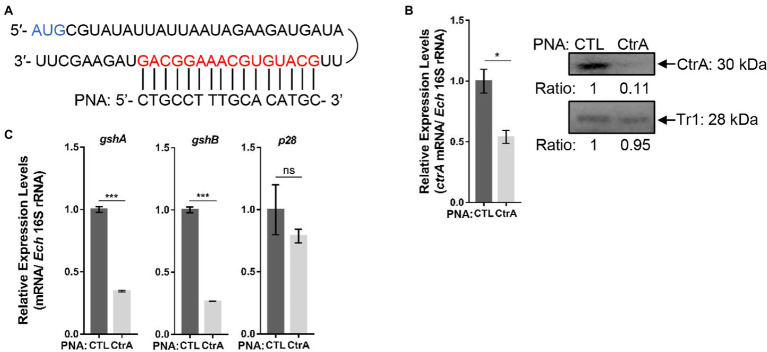
CtrA peptide nucleic acid (PNA) transfection inhibits the expression of *ctrA*, *gshA*, and *gshB.* **(A)** CtrA PNA targets the *ctrA* mRNA. The mRNA sequence of *ctrA* from the translation start codon (AUG) and the CtrA PNA sequence are shown. **(B)** CtrA PNA significantly reduces *E. chaffeensis ctrA* mRNA and CtrA protein levels. The *ctrA* mRNA levels were determined with qRT-PCR and normalized against those of *E. chaffeensis* 16S rRNA (left). Relative values to the amount of CTL PNA-transfected *E. chaffeensis* are shown. Data indicate means ± standard deviations (*n* = 3). The significant differences are represented by *p*-values determined with Student’s *t*-test (^*^p < 0.05). The protein levels of CtrA or Tr1 (negative control) were determined with Western blotting (right). The numbers below the panels indicate the relative intensity of each protein band. The protein level of CTL PNA-transfected *E. chaffeensis* is set as 1. **(C)** CtrA PNA significantly reduces the expression of *gshA* and *gshB*. The expression levels of *gshA*, *gshB*, and *p28* were determined with qRT-PCR and normalized against those of bacteria 16S rRNA. Relative values to the amount of CTL PNA-transfected *E. chaffeensis* are shown. Data indicate means ± standard deviations (*n* = 3). The significant differences are represented by *p*-values determined with Student’s *t*-test (ns indicates *p* > 0.05, ^***^p < 0.001).

PNA is a DNA mimic that has been shown to bind single- and double-stranded DNA and RNA with high affinity and specificity (Pelc et al., 2015), and inhibits transcription from double-stranded DNA (Nielsen et al., 1991) and translation from RNA (Good and Nielsen, 1999). Therefore, we designed CtrA PNA that specifically binds near the translation start site of *ctrA* (Figure 4A). Transfection of host cell-free *E. chaffeensis* with CtrA PNA significantly reduced *ctrA* mRNA level at 36 h p.i. (Figure 4B). The CtrA PNA specificity was confirmed by the results that CtrA PNA transfection significantly reduced the CtrA protein level but had no effects on the protein level of another transcription regulator, Tr1 (Figure 4B). The transfection with CtrA PNA significantly reduced the expression of *gshA* and *gshB* in *E. chaffeensis*, while it had no effects on the expression of *p28*, a gene regulated by Tr1 (Figure 4C). These results indicate that CtrA is involved in the regulation of *gshA* and *gshB* expression.

### CtrA Directly Binds to the Promoters of *gshA* and *gshB* and Activates Their Expression

**Figure 5:**
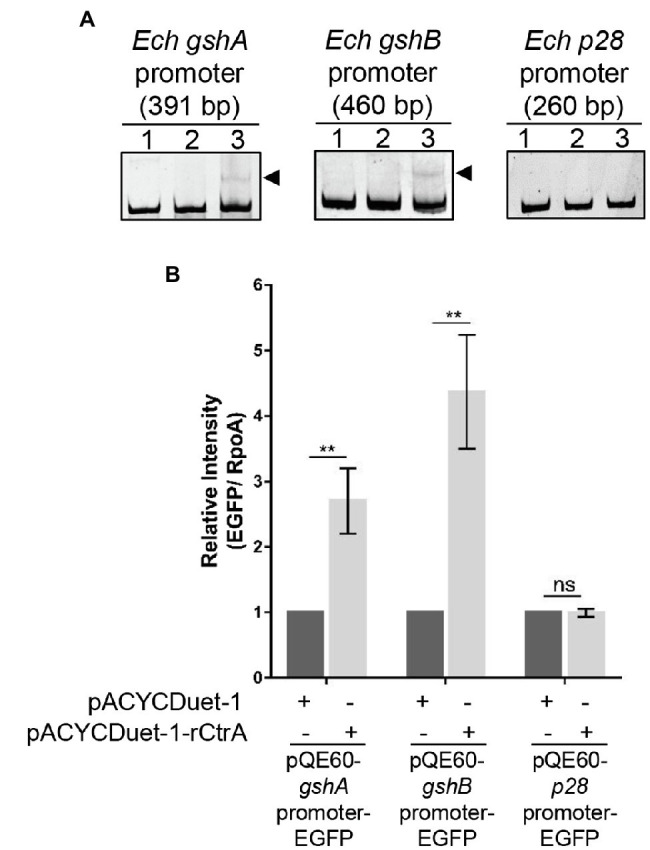
CtrA binds to the promoter regions of *gshA* and *gshB* and activates their expression. **(A)** CtrA binds to the promoter regions of *gshA* and *gshB*. DNA probe (50 ng) was incubated alone (lane 1), with GST (2.5 μM, lane 2), or with GST-rCtrA (2.5 μM, lane 3) on ice for 30 min. Shifted bands are indicated by arrowheads. The *p28* promoter is shown as a negative control. The length (bp) of the probe is shown above each panel. **(B)** CtrA activates the expression of *gshA* and *gshB*. *Escherichia coli* BL21(DE3) strains grown to an OD_600_ of 0.4 were induced to express rCtrA with 0.05 mM IPTG at 37°C for 3 h. The amount of enhanced green fluorescent protein (EGFP) or RpoA was determined using Western blotting. The relative intensities of EGFP to those of RpoA were measured by Image J and calculated by setting the value of bacteria containing pACYCDuet-1 and corresponding pQE60-promoter-EGFP fusion construct as 1. Data indicate means ± standard deviations (*n* = 3). The significant differences are represented by *p*-values determined with Student’s *t*-test (ns indicates *p* > 0.05, ^**^p < 0.01).

To investigate whether CtrA directly regulates the expression of *gshA* and *gshB*, we performed EMSA using GST-rCtrA (Cheng et al., 2011). The purified GST-rCtrA showed a single band on the SDS-PAGE gel (Supplementary Figure S4). The DNA probes derived from the promoter regions of *gshA* and *gshB* were shifted upon the incubation with GST-rCtrA (Figure 5A). The binding specificity was confirmed by the results that no shifted band was detected when the DNA probes were incubated with purified GST protein or when a DNA probe derived from the *p28* promoter region, which does not contain CtrA 8-mer or 9-mer binding motifs allowing 1-bp mismatch, was incubated with GST-rCtrA (Figure 5A). These results indicate that CtrA directly binds to the promoters of *gshA* and *gshB* in *E. chaffeensis*.

We then examined whether CtrA activates the expression of *gshA* and *gshB.* The promoter region of *gshA*, *gshB*, or *p28* was inserted upstream to the promoter-less *egfp* gene in the pQE60 plasmid to generate *gshA*-EGFP, *gshB*-EGFP, or *p28*-EGFP fusion constructs. *Escherichia coli* BL21 (DE3) strain containing pACYCDuet-1 vector harboring *E. chaffeensis ctrA* gene (pACYCDuet-1-rCtrA) or pACYCDuet-1 vector only (negative control) was transformed with the EGFP fusion constructs, respectively. The rCtrA expression induced by IPTG resulted in a significantly higher expression of EGFP in bacteria harboring *gshA*-EGFP or *gshB*-EGFP compared with the vector control, while it had no effects in bacteria harboring *p28*-EGFP (Figure 5B and Supplementary Figure S5). These results indicate that CtrA activates the expression of *gshA* and *gshB* in *E. chaffeensis*.

### Oxidative Stress Upregulates the Expression of *gshA* and *gshB via* CtrA in *Ehrlichia chaffeensis*

**Figure 6:**
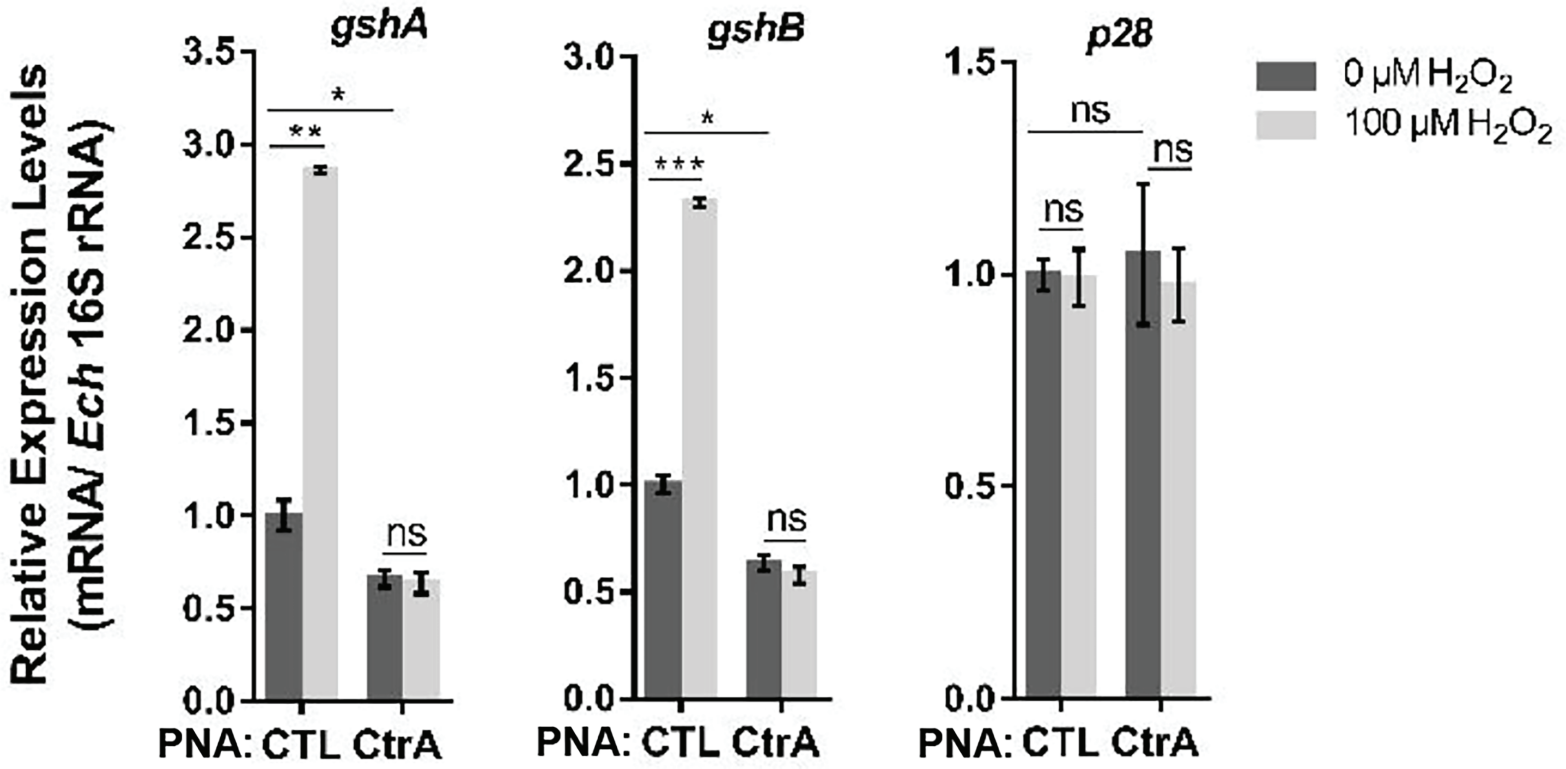
Oxidative stress upregulates the expression of *gshA* and *gshB via* CtrA in *E. chaffeensis*. At 36 h p.i. THP-1 cells synchronously infected with CTL PNA- or CtrA PNA-transfected *E. chaffeensis* were treated with H_2_O_2_ at a final concentration of 100 μM or culture medium (CTL) at 37°C for 2 h. The expression levels of *gshA*, *gshB*, and *p28* were determined with qRT-PCR and normalized against those of *E. chaffeensis* 16S rRNA. Relative values to the amount of CTL PNA-transfected *E. chaffeensis* without H_2_O_2_ treatment are shown. Data indicate means ± standard deviations (*n* = 3). The significant differences are represented by *p*-values determined with Student’s *t*-test (ns indicates *p* > 0.05, ^*^p < 0.05, ^**^p < 0.01, and ^***^p < 0.001).

We then investigated whether the upregulation of *gshA* and *gshB* upon oxidative stress is activated by CtrA. Host cell-free *E. chaffeensis* was transfected with CTL PNA or CtrA PNA, then used to infect THP-1 cells. At 36 h p.i. the infected THP-1 cells were treated with 100 μM H_2_O_2_ at 37°C for 2 h. H_2_O_2_ treatment significantly induced the expression of *gshA* and *gshB* in *E. chaffeensis* transfected with CTL PNA, while CtrA PNA transfection blocked the upregulation of *gshA* and *gshB* induced by H_2_O_2_ treatment (Figure 6). As negative controls, the *p28* expression did not change under all conditions (Figure 6). These results indicate that CtrA activates the expression of *gshA* and *gshB* upon oxidative stress.

## Discussion

Glutathione is the most abundant antioxidant molecule in cells, and it protects against oxidative stress, and maintains the intracellular redox homeostasis *via* direct and indirect interactions with ROS (Jamieson, 1998). In this study, we demonstrated that *E. chaffeensis* encodes two functional enzymes, GshA and GshB, to synthesize GSH, and the expression of *gshA* and *gshB* is upregulated upon H_2_O_2_ treatment. Thus, *E. chaffeensis* synthesizes GSH to respond to oxidative stress. *Ehrlichia chaffeensis* has evolved several mechanisms to inhibit ROS generation and reduce ROS level in infected macrophages. After *E. chaffeensis* ruptures infected macrophages for the next round of infection, it encounters ROS generated by other cells, such as neutrophils, which *E. chaffeensis* cannot inhibit (Lin and Rikihisa, 2007). The expression of *gshA* and *gshB* is upregulated at the late stage of *E. chaffeensis* intracellular growth. It is possible that *E. chaffeensis* produces GSH at this stage to protect itself from ROS generated by other types of cells when bacteria are released and start the next round of infection.

It has been reported that GSH is involved in bacterial resistance to osmotic stress in *E. coli* and *Rhizobium tropici* (Riccillo et al., 2000; Smirnova et al., 2001), and acid stress in *R. tropici*, *E. coli*, *Vibrio cholerae*, and *Lactococcus lactis* (Riccillo et al., 2000; Merrell et al., 2002; Masip et al., 2006; Zhang et al., 2007). After being released from infected macrophages, extracellular *E. chaffeensis* might encounter osmotic changes in blood or tissues, and GSH in *E. chaffeensis* might be involved in resisting environmental osmotic stress. After internalization, *E. chaffeensis* is confined within the early endosome-like membrane-bound compartments in macrophages, which retain the vacuolar type H^+^ ATPase and are slightly acidic (Barnewall et al., 1997; Rikihisa, 2015, 2022). GSH in *E. chaffeensis* might also participate in bacterial resistance to acid stress. These possibilities remain to be investigated.

Studying the functions of *E. chaffeensis* proteins is challenging due to its obligate life cycle and lack of natural plasmids. Several *E. chaffeensis* proteins have been characterized using *E. coli* mutant strains (Hang et al., 2019; Wei et al., 2021). We here employed *P. aeruginosa* mutant strains in complementation experiments and defined the functions of *E. chaffeensis* GshA and GshB. *Pseudomonas aeruginosa* is a Gram-negative opportunistic pathogenic bacterium capable of infecting humans and causing severe pulmonary disease (Gellatly and Hancock, 2013). During infection, *P. aeruginosa* interacts with the host immune system, which suggests that *P. aeruginosa* could be a good surrogate system to study the pathogenesis of *E. chaffeensis* proteins.

We found that CtrA upregulated the expression of *gshA* and *gshB* upon oxidative stress. However, the mechanism by which the oxidative stress signal is transmitted to CtrA remains to be investigated. The kinase/phosphatase activity of CckA, the cognate histidine kinase of CtrA, is regulated by the interaction with DivK and DivL or the binding of its PAS domain to cyclic-di-GMP in *Caulobacter crescentus* (Iniesta et al., 2010; Tsokos et al., 2011; Mann et al., 2016). In *E. chaffeensis*, CckA does not contain PAS domains, and DivK and DivL are missing (Cheng et al., 2006, 2011). *Ehrlichia chaffeensis* encodes two other TCSs, NtrY/NtrX and PleC/PleD (Cheng et al., 2006; Kumagai et al., 2006). It has been reported that the NtrY/NtrX system senses redox changes in *Brucella abortus* (Carrica Mdel et al., 2012). The PleC/PleD system regulates the level of cyclic-di-GMP in *E. chaffeensis*. During *E. chaffeensis* intracellular growth, NtrY/NtrX and PleC/PleD are expressed earlier than CckA/CtrA (Rikihisa, 2015). The signal of oxidative stress might be transmitted *via* NtrY/NtrX or PleC/PleD to CckA/CtrA.

Due to the reduction of bacterial genome during evolution, *E. chaffeensis* has only a few transcriptional regulators in its genome, which results in merging of genes, especially virulent genes, into the regulons of these regulators. Screening CtrA consensus binding motifs helped identify *bolA*, *ompA*, and *surE*, which are important for *E. chaffeensis* infection and intracellular survival, as the downstream genes of CtrA (Cheng et al., 2011). Here, also by screening CtrA consensus binding motifs, we determined that CtrA regulates the expression of *gshA* and *gshB*. The consensus binding motifs of CtrA may be helpful to screen more CtrA downstream genes and understand how *Ehrlichia* harness gene expression for stress resistance and the adaptation to host immune responses.

## Data Availability Statement

The original contributions presented in the study are included in the article/Supplementary Material, further inquiries can be directed to the corresponding author.

## Author Contributions

ZC and JY conceived and designed the experiments and wrote the manuscript. JY, QL, ZC, ND, XL, YL, NY, and ML performed the experiments. JY, YJ, FB, and WW analyzed the data. All authors contributed to the article and approved the submitted version.

## Funding

This work was supported by the National Science Foundation of China (32170199, 31970179, 32170177, 31870130, and 82061148018) and National Key Research and Development Project of China (2021YFE0201300). The funders had no role in the study design, data collection and interpretation, or the decision to submit the work for publication.

## Conflict of Interest

The authors declare that the research was conducted in the absence of any commercial or financial relationships that could be construed as a potential conflict of interest.

## Publisher’s Note

All claims expressed in this article are solely those of the authors and do not necessarily represent those of their affiliated organizations, or those of the publisher, the editors and the reviewers. Any product that may be evaluated in this article, or claim that may be made by its manufacturer, is not guaranteed or endorsed by the publisher.
