# Supplementary figures and images for: Glutathione Synthesis Regulated by CtrA Protects Ehrlichia chaffeensis From Host Cell Oxidative Stress
Source: Front Microbiol. 2022 Mar 30;13:846488. doi: 10.3389/fmicb.2022.846488 (PMC9005958; doi:10.3389/fmicb.2022.846488)

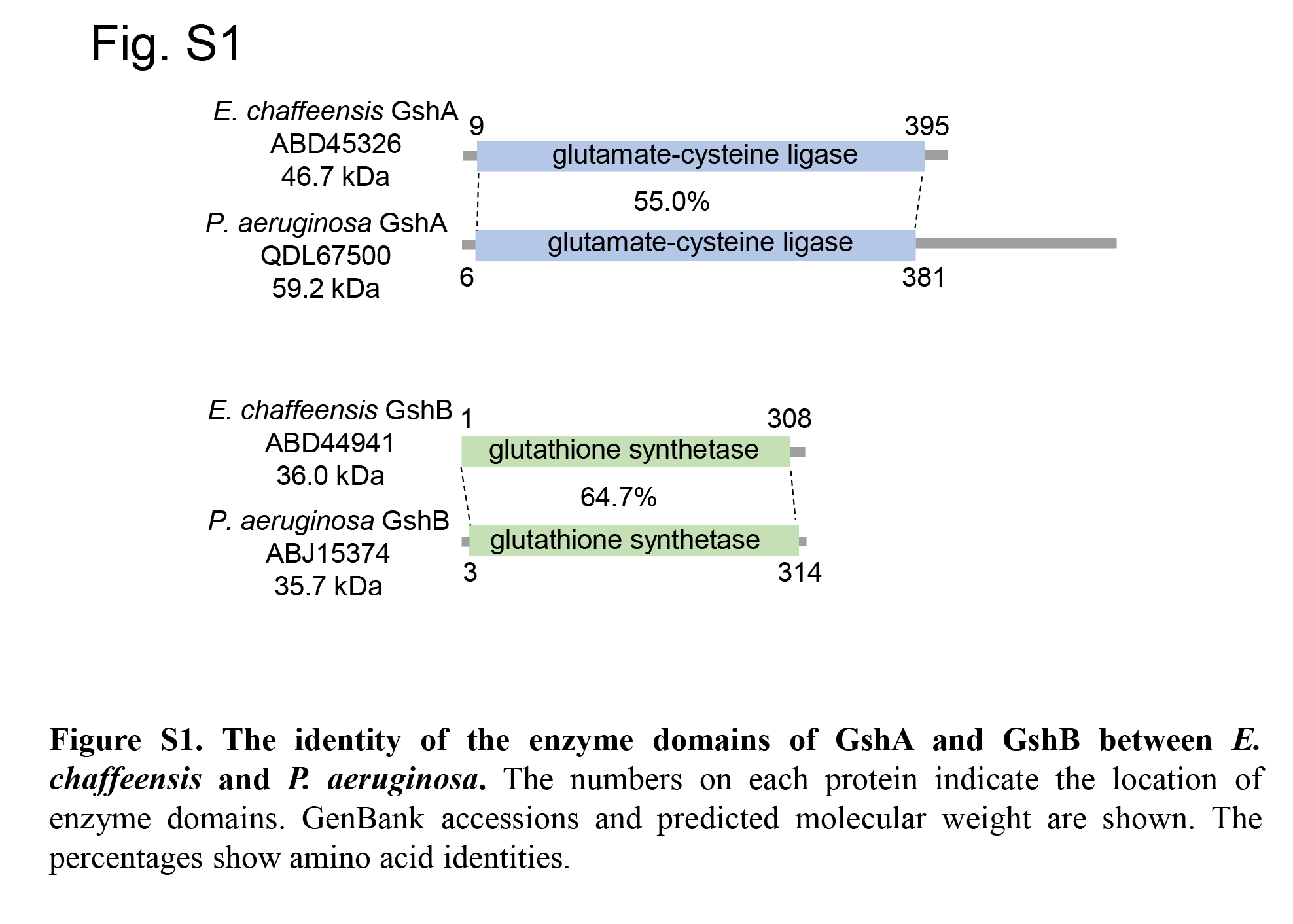

Supplement: Supplementary file 1 [file Image_1.TIF]

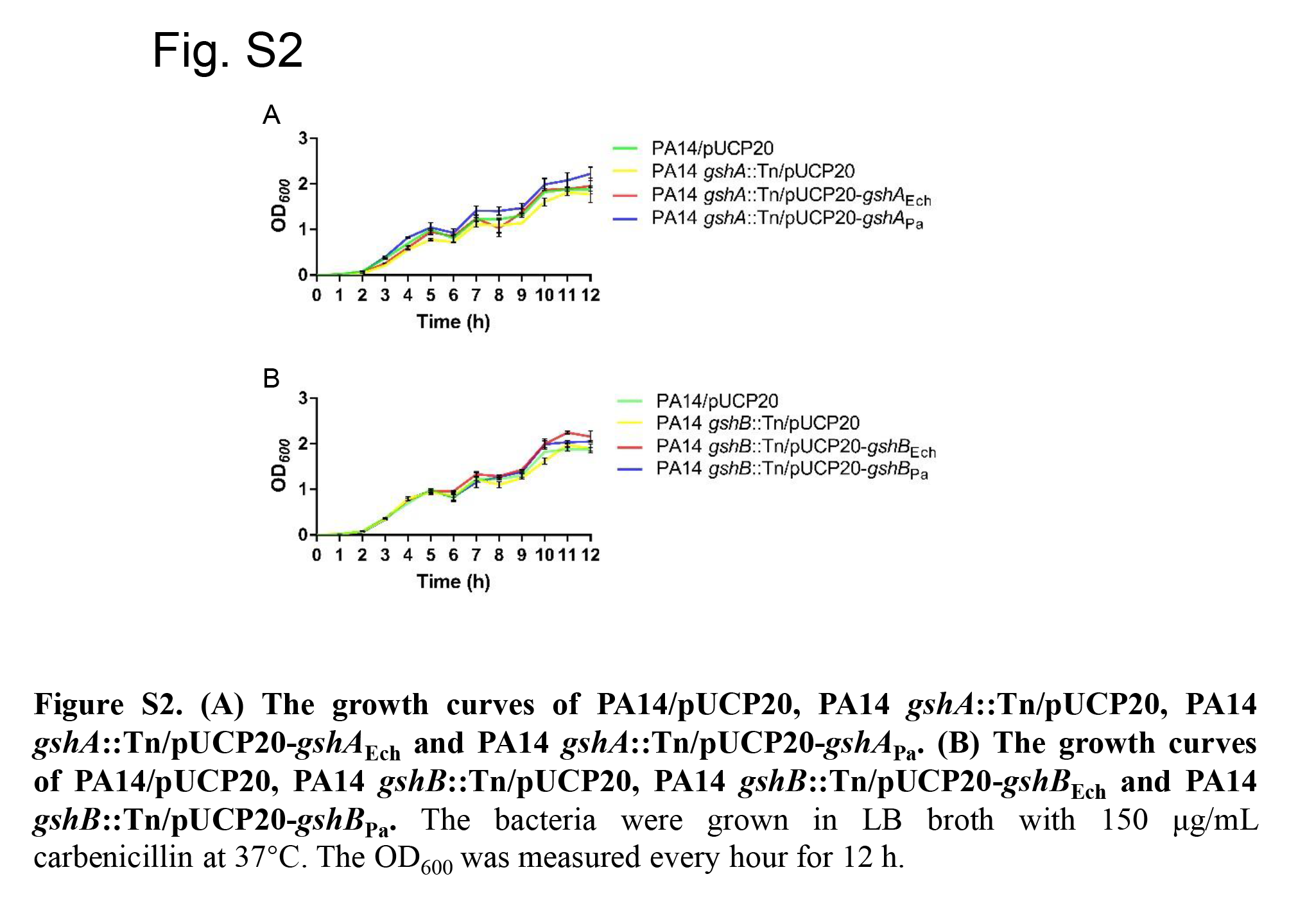

Supplement: Supplementary file 2 [file Image_2.TIF]

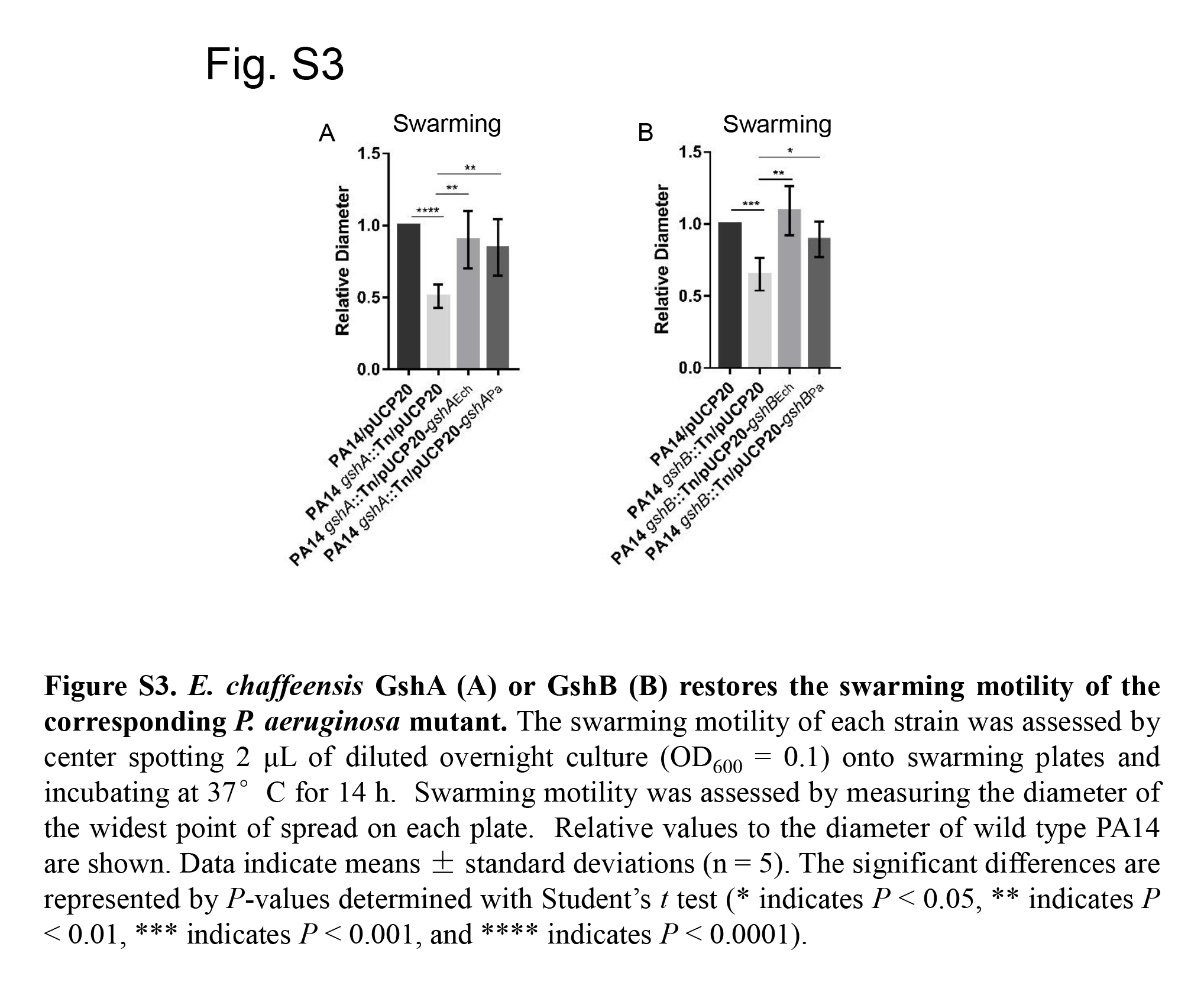

Supplement: Supplementary file 3 [file Image_3.TIF]

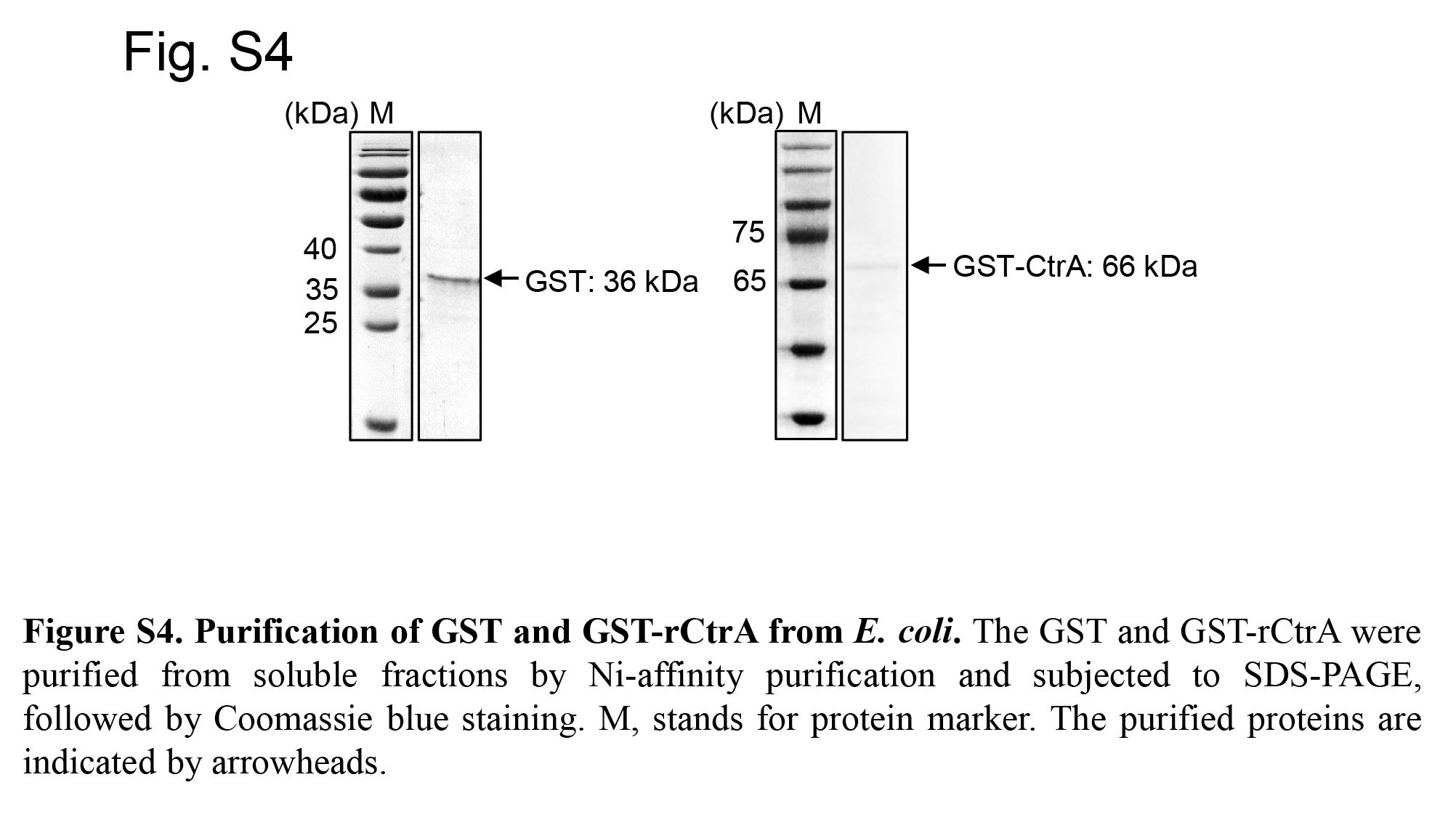

Supplement: Supplementary file 4 [file Image_4.TIF]

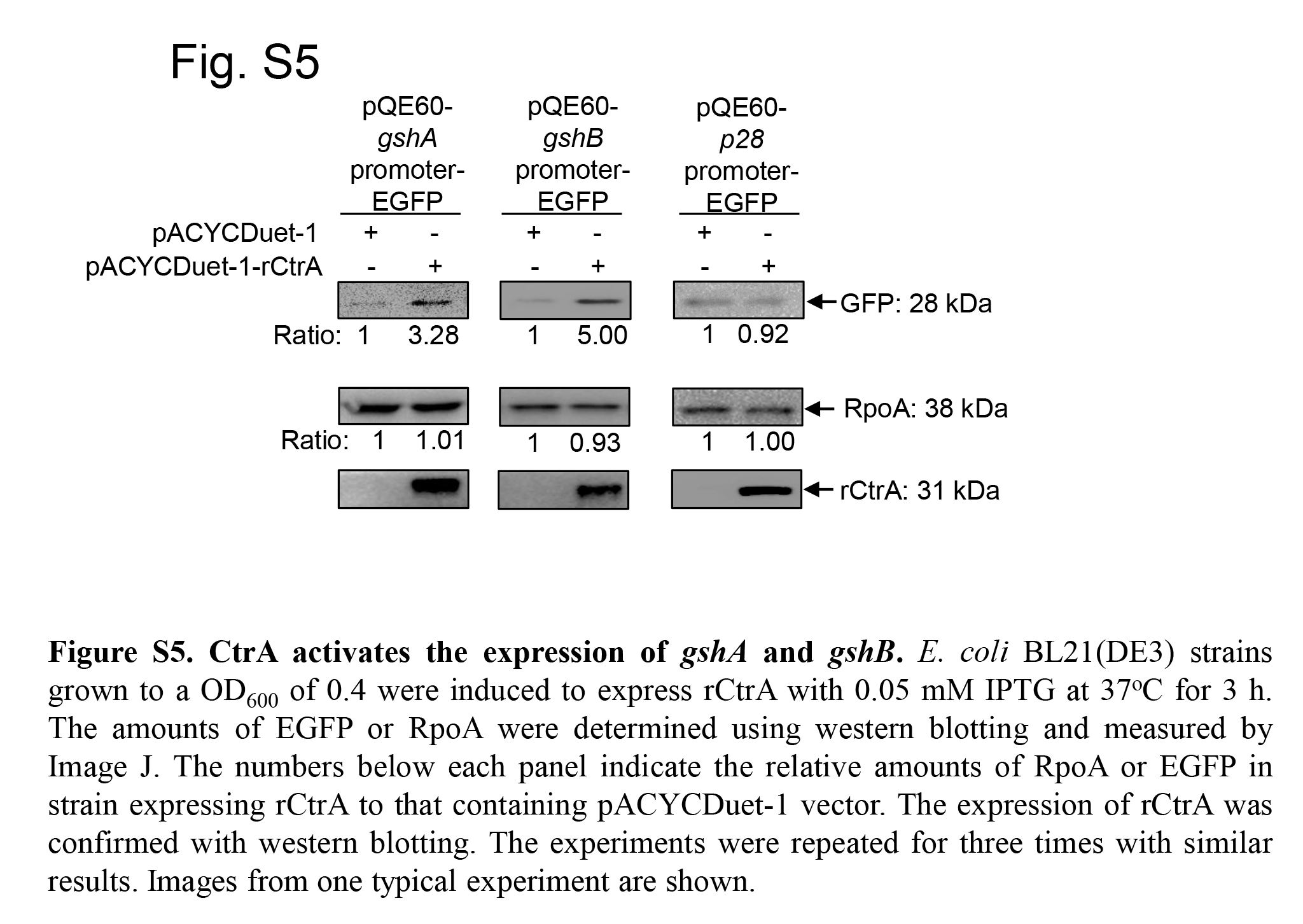

Supplement: Supplementary file 5 [file Image_5.TIF]
