# Supplementary material for: Glutathione Synthesis Regulated by CtrA Protects Ehrlichia chaffeensis From Host Cell Oxidative Stress
Source: Front Microbiol. 2022 Mar 30;13:846488. doi: 10.3389/fmicb.2022.846488 (PMC9005958; doi:10.3389/fmicb.2022.846488)
Supplement: Supplementary file 6 [file Table_1.DOCX]

**Table S1. Bacterial strains, plasmids and primers used in this study.**

| **Strain/ Plasmid /Primer** | **Description** | **Source (Reference)** |
| --- | --- | --- |
| 1. ***coli* strains** | | |
| BL21(DE3)/GST | BL21(DE3) expressing GST; Kan^r^ | This study |
| BL21(DE3)/rCtrA | BL21(DE3) expressing rCtrA; Kan^r^ | This study |
| BL21(DE3)/  pACYCDuet-1 | BL21(DE3) expressing; Chl^r^ | This study |
| BL21(DE3)/  pACYCDuet-1-rCtrA | BL21(DE3) expressing rCtrA; Chl^r^ | This study |
| DH5α/pQE60-*gshA* promoter-EGFP | DH5α / pQE60-EGFP containing *gshA* promoter; Amp^r^ | This study |
| DH5α/pQE60-*gshB* promoter-EGFP | DH5α / pQE60-EGFP containing *gshB* promoter; Amp^r^ | This study |
| DH5α/pQE60-*P28* promoter-EGFP | DH5α / pQE60-EGFP containing *p28* promoter; Amp^r^ | This study |
| BL21(DE3)/ pACYCDuet-1/ *gshA* | BL21(DE3) harboring pACYCDuet-1 and pQE60-*gshA* promoter-EGFP plasmids; Chl^r^, Amp^r^ | This study |
| BL21(DE3)/ pACYCDuet-1/*gshB* | BL21(DE3) harboring pACYCDuet-1 and pQE60-*gshB* promoter-EGFP plasmids; Chl^r^, Amp^r^ | This study |
| BL21(DE3)/ pACYCDuet-1/*p28* | BL21(DE3) harboring pACYCDuet-1 and pQE60-*p28* promoter-EGFP plasmids; Chl^r^, Amp^r^ | This study |
| BL21(DE3)/ pACYCDuet-1-rCtrA / *gshA* | BL21(DE3) harboring pACYCDuet-1-rCtrA and pQE60-*gshA* promoter-EGFP plasmids; Chl^r^, Amp^r^ | This study |
| BL21(DE3)/ pACYCDuet-1-rCtrA /*gshB* | BL21(DE3) harboring pACYCDuet-1-rCtrA and pQE60-*gshB* promoter-EGFP plasmids; Chl^r^ , Amp^r^ | This study |
| BL21(DE3)/ pACYCDuet-1-rCtrA /*p28* | BL21(DE3) harboring pACYCDuet-1-rCtrA and pQE60*-p28* promoter-EGFP plasmids; Chl^r^, Amp^r^ | This study |
| ***P. aeruginosa* strains** | | |
| PA14 | Wild type *P. aeruginosa* strain | Liberati *et al*., 2006 |
| PA14 *gshA*::Tn/pUCP20 | PA14 with *gshA* disrupted by insertion of Tn harboring pUCP20 | This study |
| PA14 *gshA*::Tn/pUCP20-*gshA*_Ech_ | PA14 with *gshA* disrupted by insertion of Tn harboring pUCP20- *E. chaffeensis* *gshA* | This study |
| PA14 *gshA*::Tn/  pUCP20-*gshA*_Pa_ | PA14 with *gshA* disrupted by insertion of Tn harboring pUCP20-*P. aeruginosa* PA14 *gshA* | This study |
| PA14 *gshB*::Tn/pUCP20 | PA14 with *gshB* disrupted by insertion of Tn harboring pUCP20 | This study |
| PA14 *gshB*::Tn/  pUCP20-*gshB*_Ech_ | PA14 with *gshB* disrupted by insertion of Tn harboring pUCP20- *E. chaffeensis* *gshB* | This study |
| PA14*gshB*::Tn/  pUCP20- *gshB*_Pa_ | PA14 with *gshB* disrupted by insertion of Tn harboring pUCP20-*P. aeruginosa* PA14 *gshB* | This study |
| **Plasmid** |  |  |
| pET-41a (+) | Clone vector; Kan^r^ | Novagen |
| pCtrA | pET-41a(+) harboring *ctrA* gene; Kan^r^ | This study |
| pACYCDuet-1 | Clone vector; Chl^r^ | Novagen |
| pACYCDuet-1-rCtrA | pACYCDuet-1 harboring *ctrA* gene; Chl^r^ | This study |
| pQE60-EGFP | Amp^r^ | From Doctor Bi |
| pQE60-*gshA* promoter-EGFP | pQE60-EGFP harboring *gshA* promoter; Amp^r^ | This study |
| pQE60-*gshB* promoter-EGFP | pQE60-EGFP harboring *gshB* promoter; Amp^r^ | This study |
| pQE60-*p28* promoter-EGFP | pQE60-EGFP harboring *p28* promoter; Amp^r^ | This study |
| **Primer** | **Sequence (5’→3’)** | **Function** |
| CtrA-F | GTCCCATGGGAATGCGTATATTATTAATAGAAGATG | Protein expression |
| CtrA-R | GGCTCGAGTTATGCTTCCTCAACATACTTTTTA | Protein expression |
| pACYCDuet-1-rCtrA-F | GGGAGATCTCATGCGTATATTATTAATAGAAGA | EGFP reporter assay |
| pACYCDuet-1-rCtrA-R | CGGGGTACCTTATGCTTCCTCAACATACTTT | EGFP reporter assay |
| pQE60-*gshA* promoter*-*F | CCCCTCGAGGAACGTCTGGTTGGTCTGC | EGFP reporter assay |
| pQE60-*gshA* promoter*-*R | CGCGAATTCTGCGAGTAAACTCATAGGACA | EGFP reporter assay |
| pQE60-*gshB* promoter-F | CCCCTCGAGGAGGAGTTGGATAAGGTTGTA | EGFP reporter assay |
| pQE60-*gshB* promoter-R | CGCGAATTCATATTACCTCTTATATTTCTCTAC | EGFP reporter assay |
| pQE60-*p28* promoter-F | GGGCTCGAGtgctgcaggtaaataaaaatagt | EGFP reporter assay |
| pQE60-*p28* promoter*-*R | CCCGAATTCATATAACCTAATAGTGACAAATAAA | EGFP reporter assay |
| *gshA-*promoter*-*F | GAACGTCTGGTTGGTCTGC | EMSA |
| *gshA-*promoter*-*R | TGCGAGTAAACTCATAGGACA | EMSA |
| *gshB-*promoter-F | GAGGAGTTGGATAAGGTTGTA | EMSA |
| *gshB-*promoter-R | TCTTATAACCCTTACTTTATTGAA | EMSA |
| *p28-*promoter-F | TGCTGCAGGTAAATAAAAATAGT | EMSA |
| *p28-*promoter*-*R | ATATAACCTAATAGTGACAAATAAA | EMSA |
| *16S rRNA*-F  (*E. chaffeensis*) | GGTGAGTAATGCGTAGGAATC | qRT-PCR |
| *16S rRNA*-R  (*E. chaffeensis*) | GCTCATCTAATAGCGATAAATC | qRT-PCR |
| *gshA-*qRT-F | CGGTTGATTTAAGAGTATCTGA | qRT-PCR |
| *gshA-*qRT-R | GCTACAGTTCGCATAATTAACTA | qRT-PCR |
| *gshB*-qRT-F | ATTGCCAGAGCATGCTTGGTA | qRT-PCR |
| *gshB*-qRT-R | CAAATGGTGGGTTTTGACGCA | qRT-PCR |
| *ctrA*-qRT-F | TGCATGTGCAAAGGCAGTAG | qRT-PCR |
| *ctrA*-qRT-R | ACATCATAGTCATCATTCTTCG | qRT-PCR |
| *p28*-qRT-F | TACTCAATGGATGGTCCAAG | qRT-PCR |
| *p28*-qRT-R | TCTGCTGCTGAGTTATGGG | qRT-PCR |
| *gshA* (*E. chaffeensis*)-F | GGGGGATCCATGACGGTAATTATTGATACATTA | construct vector |
| *gshA* (*E. chaffeensis*)-R | CCCAAGCTTTTAACTTAGTAAGCAATTTTGCTC | construct vector |
| *gshB* (*E. chaffeensis*)-F | GGGGGATCCATGGCACTAATTGTTGCTTTTC | construct vector |
| *gshB* (*E. chaffeensis*)-R | CCCAAGCTTCTATATACTATTATGTTTTTCATAAA | construct vector |
| *gshA* (*P. aeruginosa*)-F | GGGGGATCCTTGAGCGATCTTCTCTCCCG | construct vector |
| *gshA* (*P. aeruginosa*)-R | CCCAAGCTTTCAGTTGCTGATCAGGCCG | construct vector |
| *gshB* (*P. aeruginosa*)-F | GGGGGATCCATGAGCGTACGCCTCGGGA | construct vector |
| *gshB* (*P. aeruginosa*)-R | CCCAAGCTTTCAGCGGGCAGCCAGCTG | construct vector |

The enzymes sites are indicated by the underline.

Kan^r^, kanamycin resistance; Amp^r^, ampicillin resistance. Chl^r^, chloramphenicol resistance
